# There is urgent need for a Global Data Resource for Antimicrobial PK/PD: CAMO-Net GDR Initiative

**DOI:** 10.1038/s41467-025-64707-1

**Published:** 2025-11-05

**Authors:** Henry Mutegeki, Alison H. Holmes, Daudi Jjingo, Ronald Galiwango, Andrew Kambugu, Nusrat Shafiq, Kerri Hill-Cawthorne, William Hope, Shampa Das

**Affiliations:** 1 The African Center of Excellence in Bioinformatics and Data Intensive Sciences, Kampala, Uganda; 2 The Infectious Diseases Institute, Makerere University, Kampala, Uganda; 3 The Fleming Initiative, Imperial College London, London, UK; 4 Centres for Antimicrobial Optimisation Network, Imperial College London, London, UK; 5 Institute of Systems, Molecular and Integrative Biology, Department of Pharmacology and Therapeutics, University of Liverpool, Liverpool, UK; 6 Postgraduate Institute of Medical Education and Research, Department of Pharmacology, Chandigarh, India

**Keywords:** Antimicrobial resistance, Pharmacokinetics, Pharmacogenetics

## Abstract

A global data resource will unify antimicrobial PK/PD data across regions, enabling open, secure, and standardised research. This initiative will help optimise antimicrobial use and combat resistance through collaborative, data-driven insights.

## Teaser

Antimicrobial resistance research is hindered by fragmented data. CAMO-Net proposes a Global Data Resource (GDR) to unify, standardize, and share data such as PK⁄PD, within a secure Trusted Research Environment, advancing clinical decision making in a global collaboration against AMR.

## No established comprehensive PK-PD database available

Antimicrobial resistance (AMR) increasingly jeopardizes global health reducing the effectiveness of antibiotics, antifungals, and other critical drugs for infectious diseases^1^. Consequences include complex clinical management, longer hospital stays, and substantial financial burdens on healthcare systems globally^2^. AMR-related mortality is rising, with over 10 million deaths globally attributed to it by 2050^3^. Identification of regimens that simultaneously maximize efficacy and prevent the emergence of resistance is essential^4^. Robust pharmacokinetic (PK) and pharmacodynamic (PD) data are critical to optimizing dosing regimens, potentially slowing the spread of AMR and improving patient outcomes.

PK/PD data is often generated during drug development and stored with a sponsor or marketing authorization holder. PK is initially obtained from healthy volunteer studies and/or patient clinical trials that are largely based in western settings, excluding real-world patients with multiple comorbidities, extremes of ages, nutritional status, polypharmacy, and representation from geographical regions with the highest AMR burden. Data from such special populations is often generated in small cohort studies post-approval and published as stand-alone reports. A review of published PK/PD data for antibiotics in the Essential Medicines List highlights gaps in data generated in populations such as patients in low-and-middle-income-settings^5^.

Generating patient PK data is costly and challenging, with limited sample numbers, so maximising derived information is paramount. Fragmentation of PK/PD research is also characterised by the employment of heterogeneous methods and storing data in isolated silos, limiting comparative analysis and reusability. Pooling of data and analysis using population PK approaches provides a way to effectively use small cohort data from real-world populations, improving identification of sources of PK variability. Overlooking this can result in underexposure or overexposure, leading to loss of efficacy or increased adverse outcomes, respectively. For antibiotics, imprecise dosing may be associated with the emergence of resistance^6^. Bringing together diverse data on one platform enables fitting of existing models to emerging data, refinement of models and improved variability estimation. The approach allows estimation of optimised dosing regimens with a high probability of target attainment across different patient populations in the wake of creeping minimum inhibitory concentrations in resistant organisms.

Several successful data platforms illustrate the benefits of integrating large-scale health data. OpenSAFELY demonstrated the power of real-time analysis of electronic health records to inform rapid public health decisions during the COVID-19 pandemic^7,8^. GISAID’s global influenza data-sharing initiative facilitated timely pathogen surveillance and vaccine development^9^. This underlines the urgent need for a similar dedicated platform tailored specifically for PK/PD data related to antimicrobial drugs.

## The proposed solution

A Global Data Resource (GDR) dedicated explicitly to antimicrobial PK/PD, organism and host genomic data and antimicrobial usage, has the potential to transform effective design of dosing regimens. Consolidating diverse PK/PD datasets within a secure and scalable platform will facilitate high-quality data sharing, synergized analytics, and evidence-based dosing recommendations. Global access to detailed data can reduce the need to repeat studies. Implementing an ISO27001-compliant Trusted Research Environment (TRE) within the GDR, with stringent anonymization protocols and role-based data access, is essential. This approach ensures patient confidentiality and regulatory compliance while enabling meaningful, high-scale analytics.

## How this will be achieved

**Fig. 1:**
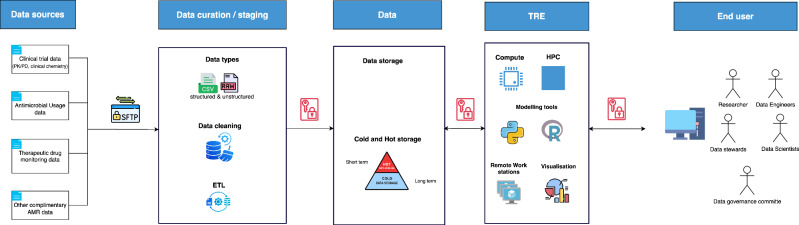
The architecture of the CAMO-Net GDR. Input will be three core AMR data streams including clinical trial data (e.g., PK, PD, genomics, clinical outcome, demographics, clinical chemistry), routine therapeutic drug monitoring data (PK data and limited demographics) and antimicrobial usage data including (demographics, dosage update, costs), all in varying file types such as comma-separated values (CSV) and fast-all (FASTA) and geographical sources. Data files shall be de-identified and securely sent over the internet through file transfer clients that utilize the encrypted secure file transfer protocol (SFTP) tunnel and arrive in a locked staging environment. There, an automated extract-transform-load (ETL) pipeline like Apache NiFi or Informatica will execute a three-step process. I. Pull raw files and attach provenance tags to enable data tracking and lineage as successfully used in the All of Us Research Program^19^. II. Tidy header names, converting units to a common scale (E.g., MIC µg/mL to mg/L), and map each field to the Observational Medical Outcomes Partnership (OMOP) common-data model vocabularies like RxNorm and Systematized Nomenclature of Medicine Clinical Terms (SNOMED) that have seen lots of success in the Observational Health Data Science and informatics (OHDSI) network^20^ III. Load the cleaned rows into a versioned structured query language (SQL) database or object storage. A tiered storage using “hot” and “cold” space will house the SQL databases and data mats in a data warehouse, similar to the UK Biobank^10^. Data will then flow into the Trusted Research Environment (TRE), comprising open-source modelling and analysis tools like R, Python or Pmetrics powered by a High Performance Computing (HPC) infrastructure. Figure 1 Icons sourced from draw.io (Apache 2.0 license) and Flaticon (www.flaticon.com, author: Freepik).

Wellcome Trust funded Centers for Antimicrobial Optimization Network |(CAMO | -NET), propose a unified GDR (Fig. 1) harmonizing data curation from various regions, enabling researchers, clinicians, and policymakers to leverage a consolidated knowledge base that captures microbial and population variability, as well as diverse clinical settings. By ensuring standardized procedures for data ingestion, quality assurance, analysis, and sharing, the GDR aims to reduce duplicative research efforts and accelerate the dissemination of best practices in antimicrobial use.

Regional hubs often use different data formats and collection standards, necessitating substantial upfront efforts to standardize and facilitate meaningful cross-comparison. For all incoming data, guidance templates for data collection and language standardization outlining required variable names, units, and coding rules will be generated in advance ensuring all uploaded data fits the set framework. Population PK analyses will be expected to adhere to guidelines issued by the European Medicines Agency^11^ and the U.S. Food and Drug Administration^12^ detailing the expected diagnostics, model evaluation steps and selection standards. Anchoring every analysis to these guidelines will keep PK results consistent and comparable across all contributing studies.

A comprehensive data governance framework will ensure effective data management, security, and quality, while safeguarding patient privacy. Compliance with the varying international and national regulations will be achieved, guided by ISO27001 and GDPR compliance^13^. Robust anonymization protocols, clearly defined international data-sharing agreements, and compliance with regional laws will all be applied^14^. A multi-national Advisory and Oversight Committee will be established to oversee data integrity, ethical concerns, and regulatory adherence. This multidisciplinary committee will comprise clinicians, scientists, data stewards, engineers, cybersecurity officers, and legal experts, with clearly articulated roles and responsibilities. The framework will also establish key policies on data privacy, sharing, use, and quality standards. Oversight by this diverse committee will ensure a people-centred, transparent, and accountable governance process from start to finish.

Researchers will submit a concept note, listing required cohorts and variables and an outline of data outputs and publication strategy. Data stewards will match the request against the catalogue, and the committee will review feasibility, ethical fit and compliance with rules. Like the OpenSAFELY model, the committee will approve, request revision or reject within ten days. Approval will prompt data engineers to spin up a secure TRE workspace with access to de-identified and authorized data fields for the researcher, and only aggregate, disclosure-checked outputs will pass through a digital air-lock. The data governance committee will closely track publication outputs, maintaining a clear and transparent publication policy. The goal is for researchers to bring modelled results back into the GDR thus keeping the resource updated and useful for future research.

The GDR must enhance data sharing opportunities and capacity building, and improve clinical outcomes, to ensure sustained financial support and active participation from stakeholders. FAIR (Findable, Accessible, Interoperable and Reusable) principles will be at the core, establishing openness, transparency and usability across different regions, an effective strategy in other health data projects such as the AMR-X framework^15^. This includes open sourcing and sharing code, documentation and data formats to aid reuse and collaboration. Raw data, key outputs from PK/PD such as model structures, parameter estimates, simulation results and visualisations will be made available. A repository of bioanalytical validation reports and Standard Operating Procedures (SOPs) will be stored to enable consistency in the PK data. These core data and outputs will help researchers and clinicians apply insights from one setting to another.

GDR development will be supported by in-kind contribution by cloud-providers as well as an injection of investment. Beyond initial seed funding, the GDR will adopt a multi-pronged sustainability strategy involving academic institutions and global AMR initiatives, service-based revenue, and public–private partnerships. A tiered subscription model or pay-as-you-go schemes will allow advanced users to pay for enhanced services such as HPC access, R workspaces for PK/PD modelling within the TRE, supported by grants. Additional revenue will be sought through corporate sponsorships from industry stakeholders like pharmaceutical companies. Public–private partnerships, governed ethically, will help ensure both financial viability and open science integrity. Contributor recognition and training programmes will foster a vibrant, global user and curator community. This blend of user-paid resources, partnerships, royalties, and subscriptions aims to create a self-sustaining, evolving platform for global antimicrobial research supported by a diversified funding portfolio to ensure resilience, independence, and long-term impact.

## Projected impact

The GDR is poised to substantially improve antimicrobial therapy by promoting precision dosing, thereby optimizing clinical outcomes and reducing healthcare expenditures. Evidence from model-informed precision dosing strategies demonstrates tailored therapy lowering rates of treatment failure by up to 20–30% and decreasing adverse drug reactions by 15–25%, especially in high-risk groups such as neonates, the critically ill, and patients with renal or hepatic impairment^16^.

Hospital-based antimicrobial stewardship programs incorporating PK/PD tools and therapeutic drug monitoring have shown per-patient cost savings of $300 to $1000, attributed to shorter hospital stays, reduced ICU admissions, a decline in drug-related complications and fewer treatment modifications^17,18^. Such interventions benefit individual patient outcomes and also generate substantial resource efficiencies at the institutional and healthcare system levels, underscoring the dual clinical and economic value of precision antimicrobial therapy.

## The way forward

The GDR will unify antimicrobial PK/PD, AMR usage and genomic data within a secure, collaborative environment. To succeed, continued stakeholder engagement, sustainable funding mechanisms, and incentives for user adoption are crucial. PK/PD research groups and healthcare organizations are encouraged to avoid creating data silos and rather to join hands in building the GDR while promising incentives such as providing free access to open source modelling tools, delivering capacity-building initiatives, and highlighting the promise of accelerated publications or direct clinical impact.

The GDR could significantly enhance our collective ability to combat AMR, driving more effective clinical interventions, personalized treatments, and strategic healthcare policies where public health authorities will be better positioned to refine AMR strategies, clinicians can adopt more personalized dosing regimens, and pharmaceutical developers can more effectively identify candidates for new antimicrobial therapies. Consolidation and harmonization of critical datasets will enhance our immediate response to AMR and provide a robust, adaptable framework for future domain-specific data repositories, significantly contributing to the global movement towards data-driven healthcare solutions.

## Data Availability

This paper does not report new datasets.

## Code availability

No custom code was developed or used for this paper.
